# The Relationship of Disordered Eating Attitudes with Stress Level, Bone Turnover Markers, and Bone Mineral Density in Obese Adolescents

**DOI:** 10.4274/jcrpe.3794

**Published:** 2017-09-01

**Authors:** Aslı Okbay Güneş, Müjgan Alikaşifoğlu, Ezgi Şen Demirdöğen, Ethem Erginöz, Türkay Demir, Mine Kucur, Oya Ercan

**Affiliations:** 1 İstanbul University Cerrahpaşa Faculty of Medicine, Department of Pediatrics, İstanbul, Turkey; 2 İstanbul University Cerrahpaşa Faculty of Medicine, Department of Pediatrics, Division of Adolescent Medicine, İstanbul, Turkey; 3 İstanbul University Cerrahpaşa Faculty of Medicine, Department of Child and Adolescent Psychiatry, İstanbul, Turkey; 4 İstanbul University Cerrahpaşa Faculty of Medicine, Department of Public Health, İstanbul, Turkey; 5 İstanbul University Cerrahpaşa Faculty of Medicine, Department of Biochemistry, İstanbul, Turkey; 6 İstanbul University Cerrahpaşa Faculty of Medicine, Department of Pediatrics, Division of Adolescent Medicine and Endocrinology, İstanbul, Turkey

**Keywords:** adolescence, obesity, cortisol, disordered eating attitude, Stress, bone turnover, bone mineral density

## Abstract

**Objective:**

To investigate the effect of stress caused by disordered eating attitudes on bone health in obese adolescents.

**Methods:**

A cross-sectional study comprising 80 obese adolescents was performed from November 2013 to September 2014. Twenty-four-hour urinary free cortisol levels were measured as a biological marker of stress. Bone turnover was evaluated using bone-specific alkaline phosphatase, serum osteocalcin, and urinary N-telopeptide concentrations. Bone mineral density was measured using dual-energy X-ray absorptiometry. The Eating Disorder Examination Questionnaire, Dutch Eating Behavior Questionnaire, Children’s Depression Inventory, and the State-Trait Anxiety Inventory for Children were used to assess eating disorders, depression, and anxiety. Psychiatric examinations were performed for binge eating disorders.

**Results:**

In the Pearson’s correlation test, a positive correlation was found between the 24-hour urinary cortisol level and Dutch Eating Behavior Questionnaire total and restrained eating subscale scores (p<0.05 for both). In linear regression analyses, the Dutch Eating Behavior Questionnaire total and restrained eating subscale scores were found to be significant contributors for urinary cortisol level (β=1.008, p=0.035; β=2.296, p=0.014, respectively). The femoral neck areal bone mineral density was found to be significantly higher in subjects who had binge eating disorder compared with those without binge eating disorder (p=0.049).

**Conclusion:**

Despite the lack of apparent effects on bone turnover and bone mineral density in our obese adolescents at the time of the study, our results suggest that disordered eating attitudes, and especially restrained eating attitudes, might be a source of stress. Therefore, studies in this area should continue.

**What is already known on this topic?**

In the literature, there is only one study investigating the relationship between disordered eating attitudes and bone health in obese adolescents. In this study, bone mineral density was found to be lower in obese individuals who had a high level of body shape concern than other obese individuals.

**What this study adds?**

In this study, no significant correlation was found between disordered eating attitudes (except binge eating disorder) and bone mineral density. The femoral neck bone mineral density was significantly higher in subjects who had binge eating disorder compared to the ones who did not have the disorder.

## INTRODUCTION

The frequency of overweight and obesity is gradually increasing worldwide (1). According to the World Health Organization (WHO), the frequency of obesity increased more than two-fold between 1980 and 2014 (1). It is known that intake of food in obese individuals increases in the presence of negative feelings including anger, fear, boredom, anxiety, stress, and sadness (2). In addition, it has also been shown that obese individuals work hard to restrict food intake (3). The efforts of obese individuals to restrict food intake may cause excessive weight gain by leading to binge eating which has the completely opposite effect (4).

Some studies showed that restriction of food intake increased endogenous cortisol secretion by leading to stress (5,6). It has been reported that increased cortisol levels inhibit bone formation by decreasing the number of osteoblasts and their function, by stimulating osteoclastogenesis, and thus affecting bone health negatively by disrupting bone turnover (7). A limited number of studies have investigated the effect of stress caused by disordered eating attitudes on bone health and the results of these studies showed variations (8,9,10,11). In studies involving premenopausal women and adolescent girls, it was found that cognitive eating restraint did not affect cortisol levels, but negatively affected bone health (8,9). In other studies conducted on premenopausal women, it was shown that cognitive eating restraint increased cortisol levels by leading to stress, and the increased cortisol levels affected bone health negatively (10).

Adolescence is an important period in terms of skeletal development (12). The results of studies directed at understanding the effect of obesity in adolescence on bone health are variable (13,14,15). In some studies, it was found that the bone mineral content (BMC) and bone mineral density (BMD) were higher in obese children and adolescents compared with those who were not obese (13,14). In contrast, in another study, it was reported that obesity in adolescents decreased BMC and BMD (15). As far as we know, there is only one study which investigated the relationship between disordered eating attitudes and bone health in obese adolescents (11). In that study, urinary free cortisol level was found to be increased in individuals who had a high level of concern with weight compared with those who had no weight concerns and BMD was found to be lower in individuals who had a high level of body shape concern compared with those who had no body shape concern (11). Based on these findings, it can be speculated that stress caused by disordered eating attitudes in obese adolescents might negatively affect bone health by increasing endogenous cortisol secretion. In this study, we aimed to investigate the effects of disordered eating attitudes and stress on bone health in adolescent obese individuals.

## METHODS

This is a prospective cross-sectional study. Eighty adolescents aged between 11 and 18 years who were referred to Cerrahpaşa Faculty of Medicine, Department of Pediatrics, Adolescent Outpatient Clinic, between November 2013 and September 2014, considered obese according to their body mass index (BMI) values as specified by Cole et al (16) were included in the study. Subjects with a chronic disease, substance addiction, or other psychiatric disorders which could prevent compliance with the study, and those who did not use any method to lose weight in the last six months were excluded.

Ethics Committee approval was obtained for the study from the Cerrahpaşa Faculty of Medicine Clinical Research Ethics Committee (date: 12.07.2013, number: 18857). A second approval was obtained from the same Committee (date: 11.03.2014, number: 6281) for the addition of the State-Trait Anxiety Inventory for Children and psychiatric face-to-face evaluation for binge eating disorder (BED). Detailed information about the study was given to the subjects who accepted to participate in the study as well as to their parents and they were asked to sign an informed consent form.

A detailed history about the subject’s physical and psychosocial health status was taken from the adolescents and their parents.

Height and body weight measurements and physical examination of the subjects were performed by the same physician. BMI was calculated using the following formula: BMI=[weight/height^2^ (kg/m^2^)] (1). Pubertal staging was performed in accordance with the Tanner staging system (17). Testicular volume was evaluated using the Prader orchidometer in boys and recorded. Telarche in girls and a testicular volume of 4 mL in boys was considered as puberty (17).

The subjects were evaluated through a face-to-face interview by a child psychiatrist in the outpatient clinic of child psychiatry regarding the presence of BED according to the Diagnostic and Statistical Manual of Mental Disorders 5^th^ Edition diagnostic criteria. The parents were not present during the interview. Other eating attitudes of the patients were evaluated using the Eating Disorder Examination Questionnaire (EDE-Q), which was adapted to Turkish by Yucel et al (18), and the Dutch Eating Behavior Questionnaire (DEBQ), which was adapted to Turkish by Bozan et al (19).

The EDE-Q: The internal consistency of the Turkish version of this scale was found high (Cronbach α=0.93) and the Cronbach α was found as 0.70 or above for each subscale (18). The scale consists of four subscales and 28 items. The subscales measure restraint, eating concerns, shape concerns, and weight concerns, and the items evaluate eating attitudes of the individual over the last four weeks. The subjects are asked to mark one of the options ranging from 0 (never) to 6 (every day) for items 1-12 and 19-21, and from 0 (none) to 6 (significantly) for items 22-28. In order to obtain a certain subscale score, the scores of the relevant items in that subscale are added up divided by the total number of items in that subscale. The scores obtained from the four subscales are also added up and divided into the number of subscales (four) in order to calculate the total score. The scale has no cut-off point. The score obtained increases with the severity of the disordered eating attitude.

The DEBQ: The internal consistency coefficients of the whole and subscales of the Turkish version are considerably high [Cronbach α (whole scale)=0.94, emotional eating=0.97, external eating=0.90, restrained eating=0.91] (19). The scale comprises thirty-three items and three subscales which measure restrained eating, emotional eating, and external eating behaviors (19). Each item has options ranging between 0 (not at all) and 5 (frequently). In order to obtain a certain subscale score, the scores of the items related with that subscale are summed. The scores obtained from the three subscales are summed in order to calculate the total score. The scale has no cut-off point. As the score obtained increases, the severity of disordered eating attitudes increases.

Children’s Depression Inventory [(CDI), adapted to Turkish]: This method was used to assess depression as a covariate, because relevant studies in the literature that were conducted on adults showed that depression negatively affected bone health by increasing cortisol levels (20,21,22). This scale involves 27 items that can be applied to children aged between 6 and 17 years. The internal consistency of the Turkish version is high (Cronbach α=0.77) (20). The child or adolescent is asked to mark the most appropriate option for the last two weeks. Each item is given a score of 0, 1, or 2 according to the severity of the symptom. The highest score is 54. The cut-off point has been recommended as 19.

State-Trait Anxiety Inventory for Children (STAI-C): Based on studies which indicate that cortisol levels increase as the level of anxiety increases and that increased levels of anxiety affect bone health (23,24), anxiety was assessed as a covariate using STAI-C. Adaptation studies of this scale showed that it could be applied to children aged 9-16 years. The internal consistency of the Turkish version is high (Cronbach α=0.82 for state anxiety and =0.81 for trait anxiety) (25). The STAI-C can be applied to groups or individuals. The scale is composed of two subscales as state anxiety and trait anxiety, each involving 20 items. The trait anxiety subscale was used in this study. In this scale, the severity of anxiety is graded using one of the options including “almost never”, “sometimes”, and “frequently”. These options are given one, two, and three points, respectively. The possible scores range between 20 and 60, and an increase in the score expresses an increase in anxiety.

Venous blood samples were obtained from the adolescents after a 12-hour fasting period; serum bone-specific alkaline phosphatase (B-ALP) was measured using a human B-ALP ELISA kit (Hangzhou Eastbiopharm, Hangzhou, China, Cat. No: CK-E10874), serum osteocalcin (OC) was measured using an EDI OC (1-43/49)-specific ELISA kit (Epitope Diagnostics Inc., San Diego, CA, USA, KT809), 24-hour urine N-terminal telopeptide (NTx) was measured using an Osteomark NTx Urine kit (Alere Scarborough, Inc., Scarborough, ME, USA, Ref 9006), and 24-hour urine free cortisol was measured using a DRG Urinary Cortisol kit (DRG International Inc., Springfield Township, NJ, USA, EIA-2989) with an ELISA assay. The adolescents and their parents were informed about how to collect a 24-hour urine sample, discarding the first urine in the morning and collecting urine in the next 24 hours including the first urine in the next morning.

The 25-hydroxy vitamin D (25-OH vit D) level was taken as a covariate in the linear regression analyses to investigate the relationship between disordered eating attitudes and bone health. Vitamin D deficiency is reported to be more frequent in obese individuals compared with individuals of normal body weight and vitamin D deficiency is known to negatively affect bone health (26,27). The measurement was performed using electrochemiluminescence with a Cobas vitamin D total kit (Roche Diagnostics GmbH, Mannheim, Germany, Ref 05894913).

Areal BMD (aBMD) was measured in the femoral neck and lumbar 1-4 vertebral (L1-4 vertebra) area. The measurements were performed by two experienced technicians using a Hologic QDR 4500W measurement device with dual-energy X-ray absorptiometry (DEXA). The femoral neck was used in measurements because it is the most appropriate BMD measurement region to evaluate the risk of hip fracture. Moreover, the fracture prediction model of the WHO involves femoral neck BMD measurements (28). Lumbar vertebrae were used for measurements because they are one of the most common body regions where osteoporotic fractures occur (29). The z-scores of the subjects were calculated using the reference values of femoral neck and L1-4 vertebral aBMD by age and sex for healthy Turkish children and histories of fracture were interrogated (30). After taking age and sex into account, z-scores of ≤-2 indicate clinically low bone mass, and z-scores between -1 and -2 indicate that an individual is at risk for low bone mass.

The Statistical Package for Social Sciences version 21.0 was used for statistical analyses. The data were assessed for normality using visual and analytic methods. Continuous variables were defined as mean ± standard deviation and categorical variables were defined as percentages. In the comparison of continuous variables by groups, Student’s t-test was used for the variables that showed normal distribution and the Mann-Whitney U test was used in the absence of normal distribution. In the assessment of the correlations between variables, Pearson’s correlation test was used for variables with parametric distribution and Spearman’s correlation test was used for those that had non-parametric distribution.

A series of linear regressions were conducted to determine whether disordered eating attitudes, as measured by the EDE-Q and DEBQ, and BED, as assessed by clinical interview, significantly contributed to the 24-hour urinary cortisol level, bone turnover markers, and BMD. Sex, age, height, weight, pubertal stage, CDI, and STAI-C scores were considered as covariates in the aforementioned models. In addition to the variables above, 25-OH vit D level was taken as a covariate in the linear regression models examining the contribution of disordered eating attitudes to bone turnover markers and BMD. Height and weight were used, rather than BMI, because height serves as an adjustment measure for bone size. A p-value of <0.05 was considered statistically significant.

## RESULTS

Mean age of the subjects was 14.01±1.59 years. Forty-six (57.5%) of the subjects were girls. Mean BMI was found as 31.29±3.06 kg/m². The relationship of disordered eating attitudes with 24-hour urine cortisol levels, bone turnover markers, femoral neck aBMD, and L1-4 vertebral aBMD values is shown in Table 1. A positive correlation was found between the 24-hour urinary cortisol level and DEBQ total score and DEBQ restrained eating subscale score (p<0.05 for both). No significant difference was found between subjects with and without BED in terms of 24-hour urinary cortisol levels.

No significant difference was found between subjects with and without depression in terms of 24-hour urinary cortisol levels, bone marker levels, and BMDs. In Spearman’s correlation test, no significant correlation was found between the level of anxiety and 24-hour urinary cortisol levels, bone marker levels, and BMD.

In the linear regression model running for determination of the contribution of the DEBQ total score to the urine free cortisol level, the DEBQ total score (β=1.008, p=0.035) was the only significant contributor to the model (adjusted R2 =0.124), which means that an increase of one unit in the DEBQ total score led to an increase of 1.008 units in 24-hour urinary cortisol level (Table 2).

In the linear regression model running for determination of the contribution of the DEBQ restrained eating score to the urine free cortisol level, the DEBQ eating restrained score (β=2.296, p=0.014) and age (β=12.067, p=0.018) were the significant contributors to the model (change in model R2=0.143), which means that an increase of one unit in the DEBQ restrained eating subscale score led to an increase of 2.29 units in 24-hour urinary cortisol level (Table 3).

The femoral neck aBMDs of the subjects who had BED were found significantly higher compared with the femoral neck aBMDs of subjects without BED (p=0.049). Although this p-value was lower than the value which was considered as a statistical significance limit, it should be approached with suspicion, because the confidence interval values of both groups were overlapped (Table 4). In the linear regression model in which gender, age, height, weight, pubertal stage, vitamin D, CDI, and STAI-C scores were considered as covariates, the contribution of BED to femoral neck aBMD was investigated. When the beta coefficients were examined, no association was found between BED and femoral neck aBMD (p=0.896).

## DISCUSSION

In this study, we investigated the associations of disordered eating attitudes with cortisol, bone markers, and BMD. Significant associations were found between the DEBQ total score and DEBQ restrained eating subscale score. As a biological stress marker, 24-hour urinary cortisol was also found to be associated with these scores. In the literature, the results of studies that investigated the relationship between disordered eating attitudes and cortisol level were variable (5,6,8,9,10,11). In some studies conducted with pre- and postmenopausal women, cortisol levels were found to be higher in subjects who showed restrained eating attitudes compared with those without restrained eating attitudes (5,6). This finding was related with increased activation of the hypothalamo-pituitary-adrenal (HPA) axis caused by restrained eating attitudes by way of stress (5). On the other hand, some studies conducted on adult women and adolescent girls showed no such correlation (8,9). In the study of Schvey et al (11) conducted on obese adolescents, no relationship was found between cognitive eating restraint as assessed by the results of the Eating Disorder Examination interview and the 24-hour urinary cortisol levels. However, the 24-hour urinary cortisol level was found to show an increase as the level of weight concern increased. This finding was interpreted to mean that cognitive eating restraint did not cause stress in the study participants, but that it actually helped treatment because the participants were obese adolescents who were in search of treatment. It also suggested that the main source of stress in these individuals was their body weight (11). In contrast to the results reported by the above authors, the fact that we found significant associations between the increase in 24-hour urinary cortisol level and DEBQ total score and DEBQ restrained eating subscale score suggests that disordered eating attitudes (restrained, emotional, external eating), and especially restrained eating, causes an increase in endogeneous cortisol production as a the main source of stress.

In our study, no significant difference was found between the subjects with and without depression in terms of 24-hour urinary cortisol levels, bone markers, and BMD values. Depression was found to increase cortisol levels and/or negatively affect bone health in some studies conducted with adult women and adolescents (21,22,23,24). Another study in adult women failed to demonstrate any effect of depression on cortisol level and bone health (31). The investigators who found the cortisol level to be increased in individuals with depression explained this finding by increased activation of the HPA axis (21,23). In a study which reported that depression had no effect on the cortisol level and bone health, the authors related this finding to the fact that their patient group had mild or moderate depression and thus the HPA axis was not activated (31). When Mathew et al (32) reevaluated subjects ten years after they were diagnosed as having major depression in adolescence, in whom no relation was found between depression and serum cortisol level, the authors showed that the HPA was abnormal and cortisol levels were increased in those who attempted suicide. This finding suggested that factors including severity of depression, duration of depression, and an accompanying diagnosis of psychosis also had an impact on cortisol secretion (32). Schvey et al (11) conducted a study with obese adolescents and showed that depression had no effect on cortisol levels and bone health, and related this finding to the fact that the prevalence of depression was low (5.8%) in the subjects included in their study. In our study group, the prevalence of depression was considerably high (25%), but it could not be shown that depression, as a cause of stress, affected the 24-hour urinary cortisol level. This finding may be related with the fact that none of our patients were diagnosed with severe depression (need for hospitalization) or that the depression periods were short, because the subjects were in adolescence and the HPA axis was not yet activated.

In our study, no correlation was found between the level of anxiety and 24-hour urinary cortisol levels, bone markers, and BMD. In a study conducted on adults, it was found that the 24-hour urinary cortisol level increased as the level of anxiety increased, a finding interpreted as an effect of anxiety on the activity of the sympathetic nervous system (23). In a study conducted with adolescent girls, anxiety was found to negatively affect bone health (24). In contrast, another study conducted with adolescents found no correlation between the level of anxiety and cortisol, findings similar to our results. This lack of correlation was related with the fact that the effect of anxiety on the HPA axis did not develop until adulthood, similar to the relationship between depression and cortisol levels (33). Similarly, the anxiety level in our subjects probably did not affect the HPA axis and did not disrupt bone health, because they were still in their adolescent years.

In this study, no significant associations were found between disordered eating attitudes, as evaluated with EDE-Q and DEBQ, and L1-4 vertebral and femoral neck aBMD, whereas the femoral neck aBMDs of subjects who had BED were found to be significantly higher compared with those who had no BED (p=0.049). The number of studies investigating the relationship between disordered eating attitudes and bone health are limited and the results are variable (8,9,10,11,34,35). Some studies reported that restrained eating in adult women and adolescent girls disrupted bone turnover (8,9) and decreased BMD or BMC (8,9,10,34,35). In the study by Barrack et al (34) with adolescent female athletes, it was shown that restrained eating was the disordered eating attitude that negatively affected bone health to the greatest extent. In this same study, whole-body and lumbar vertebral BMD z-scores were also found to be higher in subjects who had BED compared with those without BED, a finding which is in line with our results. This finding may be attributed to the fact that these individuals consumed large quantities of food during a given period of time and this behavior, if practiced frequently, might have prevented development of chronic energy deficiency (34). No studies in the literature have investigated bone health in obese adolescents with BED. Additional studies are needed in this area to confirm the accuracy of our results. Two studies have investigated the relationship between disordered eating attitudes other than BED and bone health in obese adolescents and adults (11,35). Schvey et al (11) found no association between restrained eating attitude and bone health in obese adolescents, whereas it was reported that BMD decreased as body shape concern increased. Based on this finding, the investigators thought that psychological distress caused by body shape concern affected bone density (11). There are also studies showing that obesity increases bone mass (13,14). In one study, the BMD z-scores of obese adolescents were found to be higher compared with adolescents with a normal body weight, despite their low levels of 25-OH vit D and of physical activity (13). It was thought that leptin levels were high in obese individuals due to their high amount of biologically active adipose mass, and leptin was thought to be the cause of the increased BMD (13). In addition, higher insulin levels were reported in obese individuals compared with individuals with normal body weight; it was thought that insulin could lead to an increase in BMD through a direct anabolic effect on bone by way of bone receptors (13). In another study, it was found that bones were wider and stronger in obese adolescent boys compared with controls, and it was proposed that this was related with increased mechanical load on the bones in obese individuals and with the peripheral impact of estradiol (14). The results of our study suggest that the negative effects of disordered eating attitudes on bone health could have been masked with the bone mass increasing effect of obesity, as reported in the literature (13,14). We think that follow-up studies with larger sample sizes investigating the relationship between disordered eating attitudes and bone health in obese adolescents are needed. To date, only a limited number of studies have examined these parameters in obese adolescents and adults, and the study groups were small (11,35).

The first strength of our study is that BMD was measured using DEXA which is the most commonly used measurement technique for BMD and has the largest normal database (36). The assessment of stress with 24-hour urine cortisol levels and the fact that our study group comprised both girls and boys were other strengths of our study. Our study was a cross-sectional study, the number of participants was limited, and the study group was composed of obese adolescents who presented to our outpatient clinic in order to lose weight; these may be considered among the limitations of our study.

Despite the lack of apparent effects on bone turnover and BMD in our obese adolescents at the time of the study, our results suggest that disordered eating attitudes, and restrained eating attitude especially, might be a source of stress. Thus, studies investigating long-term bone health in obese individuals with disordered eating attitudes may be useful in this respect. In addition, screening of obese adolescents in terms of disordered eating attitudes might be recommended.

## Figures and Tables

**Table 1:**
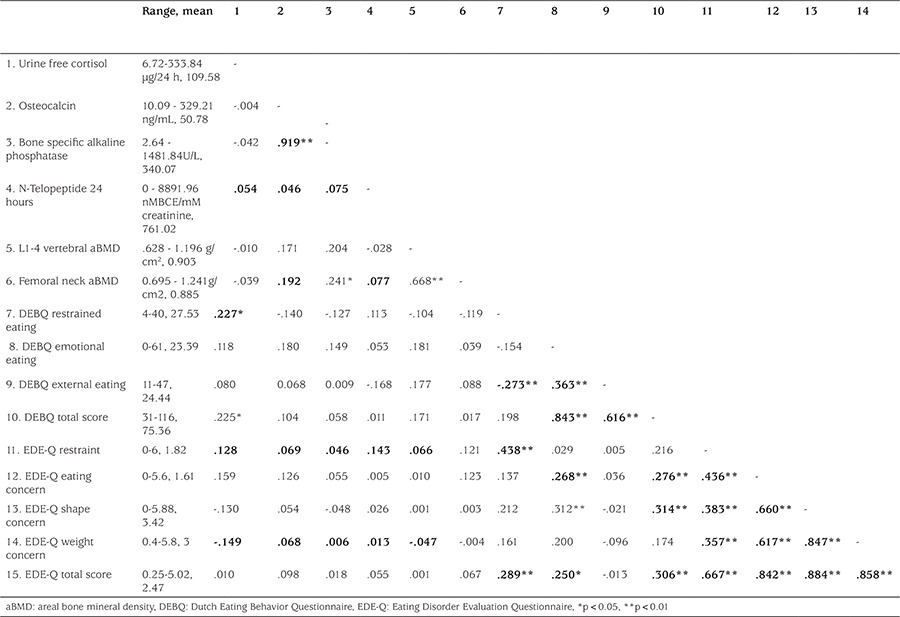
Correlations of disordered eating attitudes (measured by Dutch Eating Behavior Questionnaire and Eating Disorder Evaluation Questionnaire) with cortisol, bone turnover, and bone mineral density (Pearson’s correlation test)

**Table 2:**
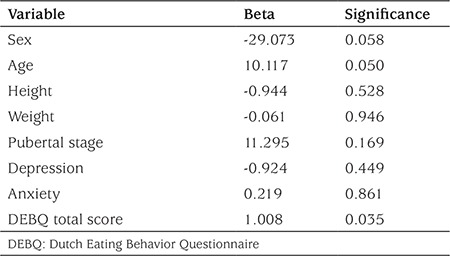
Multiple linear regression analysis: Dutch Eating Behavior Questionnaire total score and other contributors of 24-hour urine free cortisol level

**Table 3:**
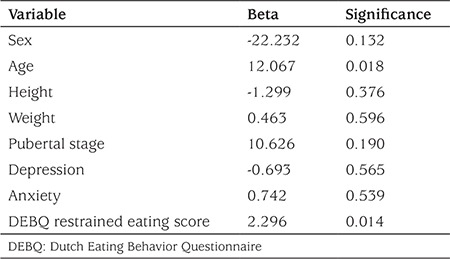
Multiple linear regression analysis: Dutch Eating Behavior Questionnaire restrained eating score and other contributors of 24-hour urine free cortisol level

**Table 4:**
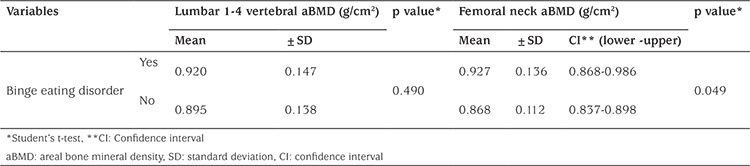
Comparison of areal bone mineral density values according to binge eating disorder status
